# Coronary by-pass for bad ventricle; adoption of "hybrid-pump" bypass

**DOI:** 10.1186/1749-8090-1-44

**Published:** 2006-11-16

**Authors:** Feza Nurözler, S Tolga Kutlu, Güngör Küçük

**Affiliations:** 1 Division of Cardiovascular Surgery, Central Hospital, İzmir, Turkey

## Abstract

**Background:**

The outcomes of on-pump and hybrid-pump bypass surgery in patients with depressed left ventricular function (EF<30%) were analyzed.

**Methods:**

109 patients with preoperative left ventricular ejection fraction of <30% and bypassable circumflex coronary disease were randomized in a double blind fashion to undergo hybrid-pump (combination of off-pump and on-pump) procedure (54 patients), or on-pump coronary bypass (55 patients). In patients who underwent hybrid-pump procedure only circumflex system was bypassed on-pump to shorten CPB and myocardial ischemic time. Pre- peri and postoperative variables were analyzed

**Results:**

Mean LVEF 24.4 ± 4.8%. The patients in hybrid-pump group received less graft than others, but difference was not significant. Duration of the surgery was not different statistically between hybrid-pump and on-pump groups. A longer intraoperative duration of ischemia and extra corporeal circulation was found in on-pump group. Significant improvement in the postoperative course such as shorter mechanical ventilation, less catecholamines and IABP usage, less ICU and hospital stay, less stroke, less need for hemodyalisis and most importantly less hospital mortality was observed in hybrid-pump group.

**Conclusion:**

Shortening the CPB and myocardial ischemic time and avoiding related problems, adoption of hybrid-pump strategy, in patients with severely impaired LVEF and bypassable circumflex coronary disease results in better outcome than conventional on-pump bypass.

## Introduction

In patients with a severely impaired left ventricular function, various modifications of the usual CABG-procedure have been published to avoid possible complications [1]. Earlier studies have concluded that risks of on-pump surgery and operative mortality are prohibitive for patients with severe left ventricular dysfunction [2-5]. Incomplete revascularization has been offered to take the advantage of a shorter ischemic time [6,7]. However the adverse effects of incomplete revascularization on survival have been particularly described for patients with an impaired left ventricular function (LVF) [8]. Off-pump coronary artery bypass surgery through a full median sternotomy has recently gained renewed interest for multi-vessel revascularization. A recent study suggested that in patients with advanced left ventricular dysfunction, the off-pump coronary artery bypass surgery has better outcomes and lowered operative mortality [9]. During the off-pump coronary artery bypass to circumflex system, exposure of the postero-lateral aspect of the heart requires displacement of the heart vertically that may cause hemodynamic instability in patients with depressed left ventricular function [9]. We have adopted the combination of off-pump and on-pump, so called "hybrid-pump" strategy that may avoid hemodynamic instability due to displacement of the heart and some of the problems associated with prolonged time on cardiopulmonary bypass.

The outcomes of on-pump and hybrid-pump bypass surgery in patients with depressed left ventricular function (EF<30%) were analyzed.

## Methods

### Patient population

Of the 1562 consecutive patients who underwent isolated coronary artery bypass graft (CABG) by the same surgeon between October 2002 and September 2005, 164 (10.5%) had preoperative left ventricular ejection fraction (LVEF) of <30%. Fifty-five of those patients with no bypassable circumflex branch coronary disease underwent off-pump coronary by-pass. Study population consisted of remaining one hundred and nine patients. All patients were advised of the procedures and their associated risks in accordance with institutional guidelines and gave informed consent. Patients were randomized in a double blind fashion to undergo hybrid-pump procedure (54 patients), or on-pump coronary bypass (55 patients). Patients undergoing concomitant valve procedure or LV aneurysmectomy were excluded. Age ranged from 44 to 76 years (mean 64). There were 76 men and 33 women. Main presentation of the patients include; congestive heart failure in 30 patients, angina pectoris in 22 patient and both in remaining. Two patient in on-pump group and three patients in hybrid-pump group were redo CABG.

Mean LVEF 24.4 ± 4.8% (range18% – 30%). The left internal mammary artery (LIMA) was used in 102 patients (95%). LIMA was anastomosed to diagonal branch in four, to circumflex system in two and to LAD in the remaining. No other arterial grafts were used due to concern about possible vasospastic response to inotropic agents postoperatively. LVEF was calculated by angiographic ventriculogram and 2-dimentional echocardiography. Heart failure was defined for the patients Class III or IV (NYHA). Renal failure was defined as a serum creatinine level above 2.0 mg/dl. Preoperatively, no patients were on hemodyalisis.

### Surgical techniques

Complete revascularization was aimed for in all patients. All operations were carried out through a full sternotomy incision. The LIMA was prepared in all cases except the reoperations in the usual fashion. In patients who underwent hybrid-pump procedure, LAD, RCA and their branches were bypassed off-pump then the patients were put on cardiopulmonary bypass (CPB) with the use of standard aortic and two-stage right atrial cannulation. Patients in hybrid-pump group were cooled to 32 C. Combination of antegrade and retrograde cold blood cardioplegia was used to achieve cardiac arrest. Circumflex system was bypassed quickly and cross-clamp removed without giving warm cardioplegia. Proximal anastomosis was performed under aortic side-biting clamp while rewarming. In on-pump group, patients were cooled to 28 C, after antegrade and retrograde induction, retrograde cold blood cardioplegia was given at the interval of 15 minutes. Warm blood cardioplegia was also given in on-pump group.

The pre-operative demographics, pre-operative co-morbidities, operative factors, pre and post-operative variables, and post-operative complications and mortality of these groups were analyzed (Table 1). Age, gender, incidence of preoperative risk factors, left ventricular ejection fraction and the degree of pre-existing angina pectoris were comparable between groups (Table 1).

**Table 1:** Preoperative parameters in groups

Groups	A	B	
	Hybrid-pump (n:54)	On-pump (n:55)	p
Age (years)	64 ± 1.4	67 ± 2.1	0.327
Gender (% male)	70	76	0.124
Reoperations (%)	5.5	3.6	0.098
Diabetes (%)	40	43	0.364
Hypertension (%)	53	58	0.213
Preoperative EF (%)	24 ± 3.4	26 ± 3.6	0.432
Angina pect (%)	68	69	0.743
Heart failure(%)	70	65	0.182
Renal failure (%)	16	18	0.464
COPD (%)	31	29	0.376

### Statistical analysis

The continuous data were presented as mean ± SD. The groups were compared using univariate analysis (X2, Fischer's exact test) and Student's unpaired t test.

## Results

Regarding intraoperative parameters in both groups, mean number of grafts per patient was 3.12 ± 0.48 in all study population, 3.08 ± 0.45 in hybrid-pump group and 3.21 ± 0.52 in on-pump group. A total of 22 endarterectomies were also performed. Ten of the endarterectomies were performed in hybrid-pump group (off-pump). Endarterectomy was undertaken on the RCA and its branches in 11 patients, the LAD in 9 and the circumflex system in 2. The patients in hybrid-pump group received less graft than others, but difference was not significant (Table 2). Duration of the surgery was not different statistically between hybrid-pump and on-pump groups. Nevertheless, a longer intraoperative duration of global ischemia was found in on-pump group compared to hybrid-pump group (7 ± 0.1 vs 22 ± 0.3, p < 0.05). There were also significant difference in duration of extracorporeal circulation between hybrid-pump and on-pump groups (21 ± 04 vs 53 ± 07, p < 0.05). Subsequently, in hybrid-pump group, the need for intraoperative use of catecholamines and IABP support was significantly less, as shown in Table 2.

**Table 2:** Intraoperative parameters in groups

Groups	A	B	
	Hybrid-pump (n:54)	On-pump (n:55)	p
Number of grafts	3.01 ± 0.45	3.23 ± 0.52	0.178
Number of endarterectomies	0.21 ± 0.06	0.23 ± 0.08	0.349
Duration of surgery (min)	164 ± 18	193 ± 20	0.238
Duration of CPB(min)	21 ± 04	53 ± 07	0.011
Duration of ischemia (min)	7 ± 01	22 ± 03	0.009
Catecholamines (%)(*adrenaline, dobutamine*)	22	40	0.032
IABP (%)	3.7	11	0.017

In hospital mortality rate were 1.8% (2 of 54) in hybrid-pump group, 5.4% (6 of 55) in on-pump group (Table 3). Cause of the deaths in hybrid-pump group were low cardiac output (1 patient) and multisystem organ failure (1 patient). In on-pump group most of the deaths were intreoperative and caused by low cardiac output (3 patients), other cause of deaths include; renal failure (2 patient) and major stroke (1 patient). Furthermore, significant differences in the postoperative course were also observed: the duration of mechanical ventilation and the ICU stay were significantly prolonged in on-pump group (Table 3). The patients in on-pump group required more inotropic and IABP support. Peak CK-levels and the MB-fraction were comparable between the hybrid-pump and on-pump groups. Additionally, no significant differences concerning ischemic changes or arrhythmias in the electrocardiography registrations or perioperative myocardial infarctions between groups were detected. Regarding ejection fraction in the postoperative course, significantly less improvement was observed in on-pump group. A significant difference in incidence of hemodyalisis, stroke and hospital mortality were also found in favor of hybrid-pump group (Table 3).

**Table 3:** Postoperative parameters in groups

Groups	A	B	
	Hybrid-pump (n:54)	On-pump(n:55)	p
Mech. ventilation (h)	18 ± 03	26 ± 05	0.042
ICU stay(d)	2.1 ± 0.3	3.2 ± 05	0.035
Catecholamines (%) (*adrenaline, dobutamine*)	18	43	0.012
IABP (%)	3.7	11	0.017
CK (U/l) (first day)	162 ± 42	179 ± 53	0.078
CK-MB (% of CK) (first day)	18 ± 6	21 ± 7	0.112
ECG-changes (ST, Q) (%)	11	13	0.237
Arrhythmias (AF, VES) (%)	28	33	0.136
Periop. myoc. infarction (%)	3.7	3.6	0.845
LV-EF (+% compared to preop.)	12.4 ± 4.3	8.6 ± 3.1	0.034
Need for hemodyalisis (%)	3.7	7.2	0.014
Stroke(%)	1.8	5.4	0.009
Mortality in hospital (%)	1.8	5.4	0.009
Hospital stay (d)	7.4 ± 1.3	10.1 ± 3.3	0.045

## Discussion

Management of severe left ventricular dysfunction from coronary artery disease is a challenging issue. Even though these patients carry a dismal outlook with medical therapy [10], cardiologist and the surgeons are reluctant for CABG due to concerns about the high risk of surgery and limited potential benefit. However, in those patients beneficial survival results of CABG by preserving functioning muscle against future infarction and recruiting hibernating muscle have been shown [11,12]. Earlier studies have concluded that risks of on-pump surgery and operative mortality are prohibitive for patients with severe left ventricular dysfunction [2-5]. Concerning the hazardous effects of prolonged cardiopulmonary by-pass and ischemic time [13] on the depressed left ventricle and impaired other organ functions due to congestive heart failure, incomplete revascularization has been offered to take the advantage of a shorter extra corporeal and ischemic time [6,7]. It is obvious that one of the most important questions is whether to accept a longer intraoperative period of ischemia for a complete revascularization or the advantage of a shorter time of ischemia for an incomplete revascularization, with. However the primary advantage of coronary bypass surgery over percutaneous interventions is complete revascularization and adverse effects of incomplete revascularization on survival have been described particularly for patients with an impaired left ventricular function [8]. In the CASS study, a study on patients with three-vessel disease, the nonrevascularization of at least one of the three systems was considered incomplete and the proportion of patients with incomplete revascularization was found higher in group with impaired left ventricular function [14]. In patients with severely depressed left ventricular function, short-term survival might be focused; however, with incomplete revascularization approache, the worse results have been reported not only in short-term mortality, but also in long-term survival [8,14]. Therefore complete revascularization should be aimed for the surgical therapy of the combination of ischemic heart disease and advanced left ventricular dysfunction.

Off-pump coronary artery bypass surgery through a full median sternotomy has recently gained renewed interest for multi-vessel revascularization. The most attractive aspect of this concept is that cardiopulmonary bypass (CPB) with its all negative side effect including organ dysfunction can be avoided. A recent study suggested that overall comparison of operative mortality and in-hospital complications between on-pump and off-pump patients, the off-pump group has the better outcomes and lowered operative mortality when compared to the high-risk patient groups [9]. Performing the off-pump coronary artery bypass surgery, bypasses to LAD, RCA and most of their braches, and intermediary artery can be done without a major hemodynamic instability. However exposure to circumflex system and the postero-lateral branch of the RCA requires displacement the heart vertically. Displacement of the beating heart in patients with depressed left ventricular function may cause a major hemodynamic instability and death [9].

In our study population concerning the hazardous effects of prolonged cardiopulmonary by-pass and ischemic time, we tried to use CPB as short as possible. Since complete revascularization was aimed, the majority of the patients with critical and by-passable circumflex disease were operated with either on-pump or "hybrid-pump". Regarding the total number of graft there was no significant difference between on-pump and hybrid-pump groups. Comparing the on-pump group, the patients in hybrid-pump group had significantly less CPB and ischemic time although duration of surgery was not found statistically different. Consequently, significant improvement in the postoperative course such as less mechanical ventilation, less catecholamines and IABP usage, less ICU and hospital stay, less stroke, less need for hemodyalisis and most importantly less hospital mortality was observed in patients operated hybrid-pump. Additionally, those patients progressed a better improvement in ejection fraction.

The major shortcomings of this experience include:

- This study was designed on the group of patient with the combination of ischemic heart disease and advanced left ventricular dysfunction to analyze the early results of two different surgical strategies.

- Shortening the CPB and myocardial ischemic time and avoiding related problems, adoption of hybrid-pump strategy, in patients with severely impaired LVEF and bypassable circumflex coronary disease results in better outcome than conventional on-pump bypass.

- Further studies are needed to support and improve the resuls of hybrid-pump strategy.

### Limitations

Although patients were randomized in a double blind fashion This may not be enough for a double blind study since the healthcare providers were able to find hybrid or conventional through the patient charts. This could impact the result.
